# ORGANIZATIONAL FACTORS RELATED TO RESIDENT-REPORTED QUALITY OF LIFE IN NURSING HOMES

**DOI:** 10.1093/geroni/igz038.1397

**Published:** 2019-11-08

**Authors:** Franziska Zúñiga, Sabine Hahn

**Affiliations:** 1 University of Basel, Basel, Switzerland; 2 Bern University of Applied Sciences, Health Division, Applied Research & Development in Nursing, Bern, Bern, Switzerland

## Abstract

Few studies so far take a broader perspective at staffing in nursing homes (NH) including e.g. the impact of activity staff on quality outcomes. Moreover, few assess resident-reported quality of life (QoL). Examining the relationship of organizational and resident factors with QoL, we report the results from a questionnaire survey of organizational characteristics from 51 Swiss nursing homes and of structured interviews with 863 residents. Residents rated their quality of life with a single item. A logistic regression model was applied. At the organizational level, a higher number of activity staff was significantly related to QoL, while at resident level, both the possibility to select their NH and less care dependency were significant predictors. Meaningful activities as well as autonomous decision-making concerning one’s living place seem of paramount importance for residents’ better perception of their QoL. NH staff mix needs to address the possibility to offer enjoyable activities.

